# Pathologic Complete Response to Neoadjuvant Nivolumab/Ipilimumab in a Patient with Metastatic Renal Cell Carcinoma

**DOI:** 10.1155/2020/8846135

**Published:** 2020-11-01

**Authors:** Taylor C. Peak, Elena M. Fenu, Michael B. Rothberg, Christopher Y. Thomas, Ronald L. Davis, Edward A. Levine

**Affiliations:** ^1^Department of Urology, Wake Forest Baptist Medical Center, Winston-Salem, NC, USA; ^2^Department of Pathology, Wake Forest Baptist Medical Center, Winston-Salem NC, USA; ^3^Department of Medical Oncology and Hematology, Wake Forest Baptist Medical Center, Winston-Salem NC, USA; ^4^Surgical Oncology Service, Department of General Surgery, Wake Forest Baptist Medical Center, Winston-Salem, NC, USA

## Abstract

Nivolumab plus ipilimumab represents an effective combination of checkpoint inhibitors that can lead to a durable response with minimal toxicity in patients with metastatic renal cell carcinoma (mRCC). We present a case of a pathologic complete response to neoadjuvant nivolumab plus ipilimumab in a patient with a 13.9 cm left renal mass and significant retroperitoneal and iliac lymphadenopathy, classified as intermediate-risk mRCC. We discuss and review the literature on complete responses after systemic therapy and the ability to predict who has undergone a complete response in the face of residual radiographic evidence of disease.

## 1. Background

With the advent of targeted immunotherapy and checkpoint inhibitors, the treatment of metastatic renal cell carcinoma (mRCC) has undergone a paradigm shift. Only two decades ago, interleukin-2 and interferon alpha-2b were the only available options, both of which had only modest success with significant toxicity. The field has changed substantially since that time, first with tyrosine kinase inhibitors (TKIs) and now more recently with checkpoint inhibitors. In addition to advancements in targeted therapies, our understanding of prognostic factors has also progressed. The more recent International Metastatic Renal Cell Carcinoma Database Consortium (IMDC) prognostic criteria have been validated in the setting of targeted therapy and represent the most reliable tool to stratify patients for treatment [1, 2].

Of the checkpoint inhibitors developed, nivolumab represents one that has demonstrated remarkable clinical efficacy [3]. It is a monoclonal antibody that binds to and blocks the programmed cell death (PD-1) protein, preventing PDL-1 from binding and inactivating T cell activity. Ipilimumab, likewise, is a monoclonal antibody that targets cytotoxic T-lymphocyte-associated protein 4 (CTLA-4) [3]. By binding to this protein, it blocks the inhibitory signal that would otherwise turn off the cytotoxic activity of T lymphocytes. More recently with their proven role as first-line therapy for intermediate- and poor-risk mRCC, case reports are emerging that demonstrate that a complete response is possible. We report a case from our institution of a pathologic complete response to neoadjuvant nivolumab plus ipilimumab in a patient with mRCC and residual radiographic disease.

## 2. Case Presentation

**Figure 1:**
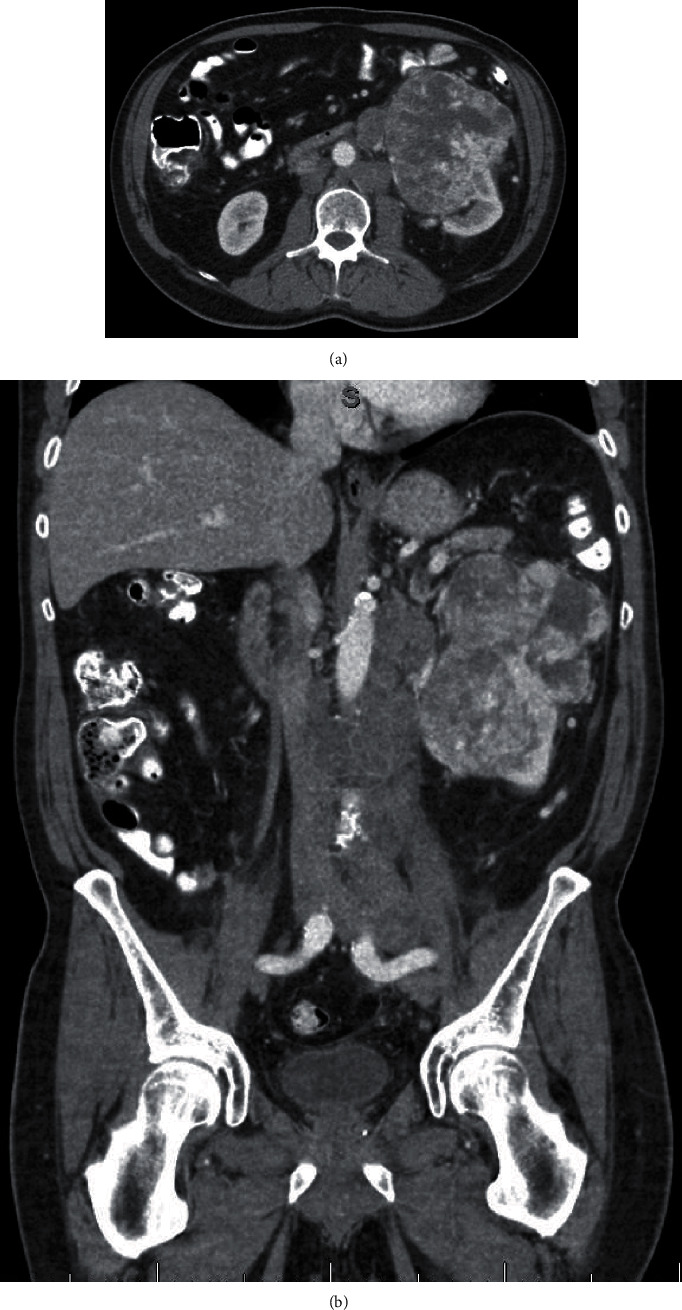
CT imaging prior to nivolumab/ipilimumab treatment: (a) axial imaging demonstrating the heterogeneously enhancing left renal mass; (b) coronal imaging demonstrating the enlarged retroperitoneal lymphadenopathy.

**Figure 2:**
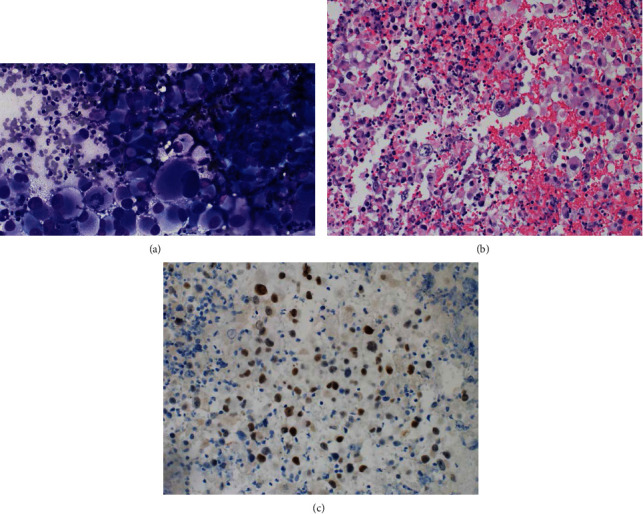
Fine needle aspiration from retroperitoneal lymph node biopsy. (a) Cytologic preparation (40x magnification) showing abundant large, pleomorphic tumor cells consistent with renal cell carcinoma. Small lymphocytes and neutrophils are present in the background for scale. (b) Cell block (20x magnification) demonstrating tumor cells with significant nuclear pleomorphism. Some cells have rhabdoid features with an eccentric nucleus and abundant eosinophilic cytoplasm. (c) PAX8 immunohistochemical staining is positive in the tumor cells, highlighting the renal origin.

**Figure 3:**
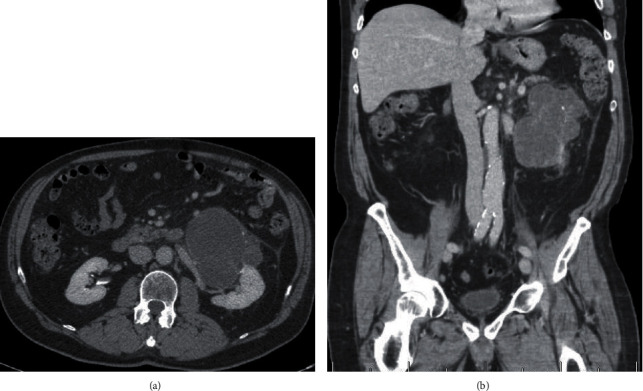
CT imaging after nivolumab/ipilimumab treatment: (a) axial imaging demonstrating significant decrease in size of the renal mass; (b) coronal imaging demonstrating resolution of the bulky retroperitoneal lymphadenopathy.

The patient is a 66-year-old man with history of hypertension, stroke, and deep vein thromboses, who initially presented in October of 2018 with a cough that was subsequently evaluated with a CT of the chest. There was a 13.9 cm incidentally noted left renal mass concerning for renal cell carcinoma (Figure 1(a)). Upon further work-up, he was noted to have significant retroperitoneal and iliac lymphadenopathy, the largest measuring 6.4 cm (Figure 1(b)). He underwent a biopsy of the retroperitoneal lymph nodes in November 2018 which was consistent with metastatic poorly differentiated renal cell carcinoma (Figures 2(a)–2(c)). Based on a performance status of <80% and eventual system therapy within one year of diagnosis, he was considered to have an IMDC risk score of 2, and as such intermediate-risk disease. He was started on nivolumab (3 mg/kg IV infused over 30 minutes) plus ipilimumab (1 mg/kg IV infused over 30 minutes) every three weeks for four cycles. This was followed up with a single agent nivolumab (240 mg IV infused) every 2 weeks for 12 cycles. There were no reported adverse events while on systemic therapy. Follow-up imaging revealed a significant decrease in disease burden with only subcentimeter retroperitoneal lymphadenopathy and shrinkage of the primary tumor to 9.5 cm (Figures 3(a) and 3(b)).

**Figure 4:**
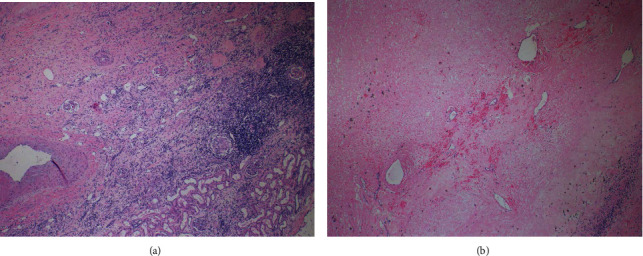
Final pathologic analyses of resected tumor specimen. (a) Resected tumor showing treatment effect (4x magnification). Large parts of the lesion are replaced by fibrosis with infiltrating lymphocytes. The relationship to normal viable kidney parenchyma is shown in the bottom right corner. (b) Resected tumor (4x magnification) demonstrating that the center of the lesion is entirely necrotic. No viable tumor cells remain.

In October of 2019, the tumor was deemed surgically resectable. He was thus taken to the operating room for a complete resection. A midline laparotomy incision was used for the intraperitoneal approach. Intraoperatively, the tail of the pancreas and spleen were both fixed to the anterior surface of Gerota's fascia, thus requiring a distal pancreatectomy and splenectomy. Inferiorly, the descending mesocolon was found encased in the fibrotic mass, necessitating ligation of the inferior mesenteric artery and resection of the mesentery. The left kidney was eventually freed of its attachments through remarkably fibrotic tissue planes and removed, en bloc with the distal pancreas and spleen. Because of the immunotherapy-induced tissue response, there was no way of differentiating grossly enlarged lymph nodes from surrounding fibrosis. The patient did well postoperatively and was discharged on postoperative day nine. Pathology of the specimen returned as having no identifiable residual tumor present, with only extensive fibrosis, necrosis, chronic inflammation, hemorrhage, and edema (Figure 4). There was one renal hilar lymph node identified in the specimen, and it was negative for tumor as well. Immunotherapy was discontinued after surgery. Follow-up imaging with CT chest, abdomen, and pelvis revealed no evidence of disease recurrence at 8 months postoperatively.

## 3. Discussion

Our case not only offers further support for the role of immunotherapy as first-line therapy for mRCC but also uniquely demonstrates that even a partial radiographic response may, in fact, represent a complete pathologic response at the time of radical nephrectomy. Obtaining such a response to systemic therapy has been an elusive goal for locally advanced or mRCC. The CheckMate 214 trial demonstrated that nivolumab plus ipilimumab led to improved overall survival in comparison to sunitinib in intermediate- and poor-risk patients with metastatic renal cell carcinoma [4]. The objective response rate was 42% versus 20% (*p* < 0.001), and the *complete* response rate was 9% versus 1% for combination nivolumab/ipilimumab and single agent sunitinib, respectively. It is important to note, however, that unlike our patient who received immunotherapy in the neoadjuvant setting, over 80% of patients in the CheckMate 214 trial had already undergone a radical nephrectomy prior to receiving immunotherapy. Additionally, another 10% of patients in the trial had undergone radiotherapy. Therefore, the majority of patients with a complete response was based on radiographic evidence of disease regression. In contrast, the case presented here, in which there is only a partial radiographic response but subsequent complete pathologic response, represents a rare clinical entity.

There are only a few case reports demonstrating a complete pathologic response after neoadjuvant immunotherapy. In Shirotake et al., a patient with an 8.5 cm left renal tumor with multiple metastases to the brain, lung, and para-aortic lymph nodes progressed on pazopanib, everolimus, and axitinib, before starting fourth-line nivolumab [5]. This resulted in a complete response that was followed by surgical resection which found a pathologic complete response with tissue remarkable for fibrotic and lymphocyte-infiltrated tissues. Ikarashi et al. reported on a patient with a 9.7 cm right renal mass with renal vein involvement, extension into the liver, and multiple lung nodules [6]. She was started on nivolumab, and repeat imaging subsequently revealed resolution of the lung metastases and a response of the primary tumor. A radical nephrectomy and partial hepatectomy were performed, with pathology revealing no viable cancer cells. In Singla et al., the authors reported on a series of 11 patients with intermediate- or poor-risk disease who received neoadjuvant nivolumab or nivolumab plus ipilimumab and subsequently underwent nephrectomy [7]. Of these patients, only one was found to be pT0 on final pathology.

With mounting data that the new immune checkpoint inhibitors can lead to a complete pathologic response, it remains unclear if there are radiographic or histologic factors that could predict this prior to undergoing a nephrectomy. Further studies, such as the PROSPER trial (EA8143), may help determine which patients could avoid a radical nephrectomy and the associated morbidity. In this phase 3 study, patients with clinical stage ≥ T2 or node-positive RCC will be randomized to either perioperative nivolumab and nephrectomy or standard nephrectomy followed by observation. From the preoperative imaging and subsequent nephrectomy specimens in the treatment arm, clinicians might learn what factors predict a complete response.

This will undoubtedly be challenging as radiographic response patterns can be quite variable for tumors treated with immunotherapy. Specifically, immunotherapies can lead to a phenomenon known as “pseudoprogression,” where there is initial radiographic progression followed by a response or stabilization [8]. Because the standard RECIST criteria would not apply to this tumor response pattern, other imaging modalities have been studied. 18F-FDG PET/CT scans, for example, have been shown to be highly accurate in predicting a response in patients treated with immunotherapy for metastatic melanoma and non-small cell lung cancer [9, 10]. Diffusion-weighted imaging may also hold promise as there is evidence that growing tumors demonstrate restricted diffusion because of high cellularity, whereas inflammation and necrosis tumors show increased diffusion because of prominent extracellular edema and decreased cellularity [11]. In the PURE-01 study, for example, patients with muscle-invasive bladder cancer were given neoadjuvant pembrolizumab and assessed with multiparametric MRI (mpMRI) prior to radical cystectomy [12]. Through their preliminary work, criteria were developed using mpMRI to accurately and reproducibly predict a complete pathologic response.

## 4. Conclusion

In conclusion, nivolumab plus ipilimumab remains the first-line therapy for patients with poor- or intermediate-risk mRCC. Even a partial radiographic response after therapy may actually represent a complete pathologic response that would obviate the need for cytoreductive surgery. However, until we have more data to confidently predict who is effectively cured in the face of residual disease on imaging, we must counsel patients on the role of cytoreductive surgery in confirming pathologic response and potentially providing improved survival.

## Data Availability

The data used to support the findings of this study are available from the corresponding author upon request.
